# A Mycobacterial Systems Resource for the Research Community

**DOI:** 10.1128/mBio.02401-20

**Published:** 2021-03-02

**Authors:** J. A. Judd, J. Canestrari, R. Clark, A. Joseph, P. Lapierre, E. Lasek-Nesselquist, M. Mir, M. Palumbo, C. Smith, M. Stone, A. Upadhyay, S. E. Wirth, R. M. Dedrick, C. G. Meier, D. A. Russell, A. Dills, E. Dove, J. Kester, I. D. Wolf, J. Zhu, E. R. Rubin, S. Fortune, G. F. Hatfull, T. A. Gray, J. T. Wade, K. M. Derbyshire

**Affiliations:** a Wadsworth Center, New York State Department of Health, Albany, New York, USA; b Department of Biological Sciences, University of Pittsburgh, Pittsburgh, Pennsylvania, USA; c Department of Immunology and Infectious Diseases, Harvard T.H. Chan School of Public Health, Boston, Massachusetts, USA; d Department of Biomedical Sciences, University at Albany, Albany, New York, USA; Washington University School of Medicine in St. Louis

**Keywords:** CRISPRi clones, *Mycobacterium*, conserved mycobacterial proteins, knockout library, protein localization

## Abstract

Functional characterization of bacterial proteins lags far behind the identification of new protein families. This is especially true for bacterial species that are more difficult to grow and genetically manipulate than model systems such as Escherichia coli and Bacillus subtilis. To facilitate functional characterization of mycobacterial proteins, we have established a Mycobacterial Systems Resource (MSR) using the model organism Mycobacterium smegmatis. This resource focuses specifically on 1,153 highly conserved core genes that are common to many mycobacterial species, including Mycobacterium tuberculosis, in order to provide the most relevant information and resources for the mycobacterial research community. The MSR includes both biological and bioinformatic resources. The biological resource includes (i) an expression plasmid library of 1,116 genes fused to a fluorescent protein for determining protein localization; (ii) a library of 569 precise deletions of nonessential genes; and (iii) a set of 843 CRISPR-interference (CRISPRi) plasmids specifically targeted to silence expression of essential core genes and genes for which a precise deletion was not obtained. The bioinformatic resource includes information about individual genes and a detailed assessment of protein localization. We anticipate that integration of these initial functional analyses and the availability of the biological resource will facilitate studies of these core proteins in many *Mycobacterium* species, including the less experimentally tractable pathogens M. abscessus, M. avium, M. kansasii, M. leprae, M. marinum, M. tuberculosis, and M. ulcerans.

## Abstract (precis)

Diseases caused by mycobacterial species result in millions of deaths per year globally, and present a substantial health and economic burden, especially in immunocompromised patients. Difficulties inherent in working with mycobacterial pathogens have hampered the development and application of high-throughput genetics that can inform genome annotations and subsequent functional assays.

## INTRODUCTION

The massive increase in sequenced bacterial genomes and their subsequent analyses have resulted in the prediction of substantial numbers of new open reading frames (ORFs). Unfortunately, the functions of many of the encoded proteins are still to be determined and functional validation is lacking for many annotated genes. In many cases, hypothetical ORFs predicted from genome sequences encode proteins that are highly conserved and, thus, are likely to play important biological roles. While traditional gene-targeted approaches can assign functions to these ORFs, more comprehensive, system-wide approaches are needed to dramatically accelerate the functional annotation of bacterial genomes, especially for less characterized non-model systems (1). The ability to integrate information from many experimental approaches (genomic, biochemical, cell physiological, and bioinformatic) to create a usable systems resource has been an incredibly powerful tool for the research community, best exemplified by those for Escherichia coli, Bacillus subtilis, and Saccharomyces cerevisiae (https://ecocyc.org/; http://www.bgsc.org/; https://www.yeastgenome.org/). In all cases, the foundation for these resources was a defined set of gene knockouts, together with plasmid clones expressing individual genes in a defined genetic background (2–6). These biological resources have greatly facilitated systematic analyses of unknown gene functions, and served as the starting point for reverse and synthetic genetic approaches. The subsequent integration of other data sets (e.g., protein-protein interactions, synthetic genetic arrays, protein localization studies, and phenotypic profiling) into these resources has further enhanced the assignment of functional annotations and provided new insights into cellular processes (7–12).

Mycobacterium tuberculosis accounts for millions of deaths per year globally, which creates a substantial health and economic burden (13). Other mycobacteria, including M. abscessus, M. avium, M. kansasii, M. leprae, and M. ulcerans, also have a substantial disease burden, especially in immunocompromised patients (14–16). A focus on M. tuberculosis research over the last 2 decades has improved the annotation of the M. tuberculosis genome, and the fidelity of the associated databases describing gene function and architecture (e.g., https://mycobrowser.epfl.ch/; http://genolist.pasteur.fr/TubercuList/; https://patricbrc.org/; https://www.wadsworth.org/research/scientific-resources/interactive-genomics). However, the inherent difficulties in working with this slow-growing pathogen have hampered the application of high-throughput genetics to improve genome annotations and other functional assays. Indeed, ∼30% of M. tuberculosis genes are annotated as hypothetical (17, 18).

The experimental limitations of M. tuberculosis hinder both basic and clinical research. Thousands of clinical M. tuberculosis strains have been sequenced to identify genes associated with drug resistance and virulence (19, 20). Unfortunately, the interpretation of many of these sequences is limited because so many ORFs have no known function. Transposon insertion sequencing (Tn-seq) analyses have identified genes essential for growth *in vivo* and *in vitro*, but do not address gene function (21–23). Other research efforts have resulted in the establishment of a collection of 1,289 mapped transposon insertion mutants, a library of sequence-validated nonessential M. tuberculosis gene clones in E. coli that can be used to generate targeted knockouts of M. tuberculosis genes (available from BEI Resources, https://www.beiresources.org) (24, 25), and a collection of 475 strains that allow conditional depletion of essential proteins (26). However, these resources are limited to M. tuberculosis. Most strikingly, to our knowledge, there is no centralized mutant or cloned gene resource for any other *Mycobacterium*, despite their recognized impact on global health.

**FIG 1:**
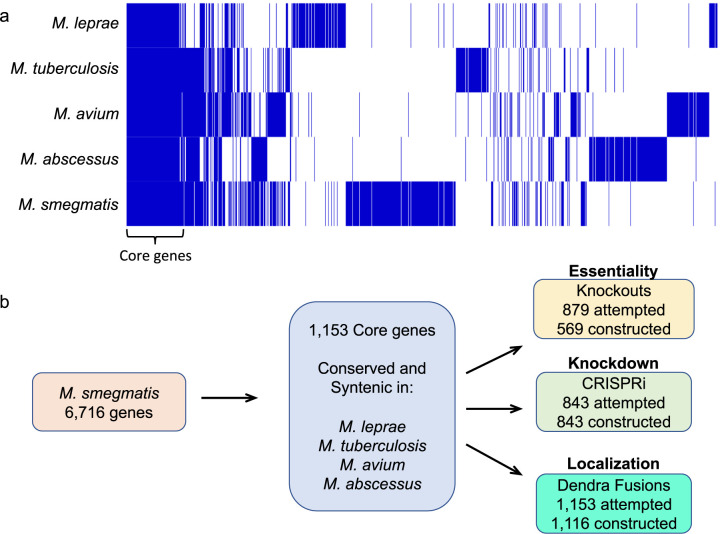
(a) A pan-genome analysis with Roary v.3.13.0 (31) identifies conserved core genes among M. smegmatis mc^2^155, M. avium 104, M. tuberculosis H37Rv, M. abscessus ATCC 19977, and M. leprae TN. Orthologs were required to share at least 50% global amino acid identity and are indicated by a vertical blue line in each genome along the *x* axis (the pan genome); white (no blue) line indicates the ortholog is absent in that genome. Approximately 15,000 genes were compared in the analysis, with the conserved core genes indicated on the left of the panel. Results were visualized with Phandango v.1.3.0 (59) and Figtree v.1.4.4 (https://github.com/rambaut/figtree). (b) Schematic representation of the MSR and the constructs generated from the core genes.

Here, we describe the construction of a mycobacterial systems resource (MSR) that we believe will dramatically accelerate research throughout the mycobacterial research community. The MSR uses M. smegmatis strain mc^2^155 as its model organism, while focusing exclusively on genes that are highly conserved across the mycobacterial genus. Thus, functional insights from the MSR will apply to all mycobacterial species. An added benefit of using mc^2^155 was that all derivatives constructed in the resource share an identical genetic background, thus enhancing the capacity to compare phenotypes and minimizing the potential effects of different genetic backgrounds. There are 2,821 predicted proteins with >50% global amino acid identity between M. smegmatis and M. tuberculosis. We further refined these conserved genes by requiring that they were also highly conserved among M. leprae, M. avium, and M. abscessus. This generated a set of 1,153 genes that share >50% amino acid identity across all these species, which form the basis of the MSR (Fig. 1a; Table S1 in the supplemental material). Our goal is not only to supplement research on M. tuberculosis, but also to enhance studies on the other important mycobacterial pathogens for which no resource or model system is currently available. Below, we present the rationale behind the MSR collection, describe the biological and data resources created, and provide proof-of-principle examples of how this resource can be utilized (Fig. 1b).

10.1128/mBio.02401-20.5 TABLE S1 Master list of 1,153 MSR genes with orthologs of >50% amino acid identity to M. tuberculosis, M. avium, M. leprae, and M. abscessus. The list includes gene, name, coordinates, M. tuberculosis H37Rv ortholog, protein length, and availability of Dendra gene fusion, gene knockout, and CRISPRi sgRNA clone. A list of genes annotated as pseudogenes in some databases is also included; the majority of these encode full-length proteins in the MSR collection. Download Table S1, XLSX file, 0.2 MB. Copyright © 2021 Judd et al. 2021 Judd et al. https://creativecommons.org/licenses/by/4.0/ This content is distributed under the terms of the Creative Commons Attribution 4.0 International license.

## RESULTS

### Rationale for selection of a core set of M. smegmatis genes.

The genome of M. smegmatis is larger than those of the pathogenic species (6.9 Mb versus 4.4 Mb for M. tuberculosis), likely reflecting the more varied demands of its environmental niche. Therefore, we have focused on the most highly conserved proteins in the M. tuberculosis complex (MTBC), M. avium, M. abscessus, and M. leprae, since we predict that these proteins regulate and/or perform shared fundamental cellular processes. Such conserved genes are best studied in the most experimentally tractable mycobacterium, M. smegmatis, providing experimental insights applicable to the pathogenic species. M. smegmatis is an ideal host for this core-gene resource because it is a nonpathogenic, fast-growing model organism with developed genetic tools suitable for high-throughput analyses (27). The virtues of M. smegmatis as a model organism are further underscored from Tn-seq analyses, which indicate that 96% of essential and growth-impaired genes in M. smegmatis have a mutual ortholog in M. tuberculosis and the majority of those (90%) are essential in M. tuberculosis (28).

The 1,153 M. smegmatis protein-coding genes were selected using a best pairwise BLAST approach (<E^−30^) with M. tuberculosis, M. leprae, M. abscessus, and M. avium genomes, with a secondary screen for synteny using MUMmer and DAGchainer (29, 30), which identifies genes that are in homologous syntenic groupings (Table S1). Similar results were obtained with independent algorithms such as Roary (Fig. 1a) (31) and OrtholugeDB, which measures phylogenetic distance ratios to predict orthologs (32, 33). The OrtholugeDB analysis confirmed that most of these genes have evident orthologs in many other sequenced mycobacterial genomes, beyond those we initially selected (Table S2). Even our lower ranked proteins, sharing over 50% identity across all the species listed, have high identity in pairwise comparisons, e.g., MSMEG_0004 (DciA) is 73% identical and 84% similar between M. smegmatis and M. tuberculosis (Fig. S1) (34). Broad conservation underscores the conserved function and, therefore, the relevance of this core gene resource for pan-mycobacterial studies. We note that the inclusion of M. leprae, which has undergone massive gene decay, reduces the overall number of conserved genes by about 600 genes, and further focuses the resource on proteins mediating fundamental core processes common to all of the species.

10.1128/mBio.02401-20.1 FIG S1 Amino acid sequence alignment of MSMEG_0004 (DciA) with homologs from M. leprae, M. abscessus, M. avium, M. marinum and M. tuberculosis. The full-length protein sequence is shown. Over 50% of the residues are identical across all six species; these residues are highlighted in yellow. MSMEG_0004 is among the lower 5% of proteins in our list of highly conserved proteins, when ranked by sequence identity. Download FIG S1, TIF file, 0.8 MB. Copyright © 2021 Judd et al. 2021 Judd et al. https://creativecommons.org/licenses/by/4.0/ This content is distributed under the terms of the Creative Commons Attribution 4.0 International license.

10.1128/mBio.02401-20.6 TABLE S2 List of orthologs determined by Ortholuge (32, 33) for M. avium, M. leprae, M. abscessus, M. marinum, and M. tuberculosis. Download Table S2, XLSX file, 0.1 MB. Copyright © 2021 Judd et al. 2021 Judd et al. https://creativecommons.org/licenses/by/4.0/ This content is distributed under the terms of the Creative Commons Attribution 4.0 International license.

Many mycobacterial genes are misannotated because existing algorithms cannot accurately predict gene features in their G/C rich genomes and because ∼1/3 of mycobacterial genes are encoded from leaderless transcripts (35, 36). To ensure accurate gene cloning and precise disruption of genes, we utilized our previously published data that integrate both transcriptome sequencing (RNA-seq) and ribosome profiling (Ribo-seq) to accurately predict gene starts (https://www.wadsworth.org/research/scientific-resources/interactive-genomics) (36). Of the 1,153 genes within the MSR, 17 are annotated as pseudogenes in Mycobrowser because they contain nucleotide changes resulting in a frameshift, which are described as “not sequencing errors.” Sequence analysis of our individual plasmid clones shows that only three of these genes (*MSMEG_2348*, *MSMEG_3479*, and *MSMEG_3831*) are pseudogenes in our strain (Table S1). The remaining 14 genes encode full-length proteins (with the exception of *MSMEG_4466*, which uses an initiation codon 15 amino acids downstream of the misannotated start). This provides a cautionary note, indicating there are distinct genetic lineages among the laboratory strains of mc^2^155. Many of these pseudogenes are predicted to be essential in both M. smegmatis and M. tuberculosis (e.g., *MSMEG_1400* encodes elongation factor G), which suggests that many of the reported sequence differences might not be correct and/or that the pseudogene-containing strains contain suppressor mutations.

### Generation of a targeted knockout library.

All conserved coding genes not identified as essential by Tn-seq were targeted for precise deletion by mycobacterial recombineering (28, 37). A high-throughput *in vitro* approach was developed to streamline the process of generating substrates for recombineering. This circumvented the need to generate cloned plasmid intermediates for each target gene, and it avoided complications presented by restriction sites present in the flanking DNA homology arms. Briefly, high-fidelity PCR was used to generate ∼300-bp flanking arms to the 5′ and 3′ side of each target gene, and these products were “sewn,” using overlap extension PCR, to a cassette encoding zeomycin-resistance (Zeo^r^) flanked by *loxP* sites (Fig. S2). To reduce the chance of polar effects on downstream genes, the upstream and downstream flanking sequences were left intact. The *loxP* sites provide the option for precise excision of the *Zeo*^r^ gene by Cre recombinase, leaving behind a single *loxP* scar. The succession of substrate PCRs, electroporation, and colony screening PCRs were performed in matched 96-well format arrays, allowing complete sets of knockout substrates to be generated in a single day.

10.1128/mBio.02401-20.2 FIG S2 High-throughput (96-well) recombineering knockout scheme. The ∼300-bp regions flanking the target gene were amplified with the indicated primers. These “armplicons” were then PCR-sewn to a central cassette encoding zeo^r^ through overlapping universal priming site homology with the arms, generating a recombination substrate of approximately 1.2 kbp. Sewing was driven by nested primers A and D. The sewn substrate was then electroporated into M. smegmatis strain mc^2^155 and recombinants selected on medium containing zeomycin. Recombinants were confirmed by a 3-primer PCR that distinguished between on-target (Zeop × D’) and off-target (F × D’) recombination. Download FIG S2, TIF file, 0.8 MB. Copyright © 2021 Judd et al. 2021 Judd et al. https://creativecommons.org/licenses/by/4.0/ This content is distributed under the terms of the Creative Commons Attribution 4.0 International license.

A total of 879 genes were subjected to gene-replacement recombineering and, of these, 569 genes were successfully targeted after two attempts (Fig. 1b and Table S1). Those genes not successfully replaced were subsequently targeted by CRISPRi.

### Application of the deletion collection to identify genes required for biofilm formation.

**FIG 2:**
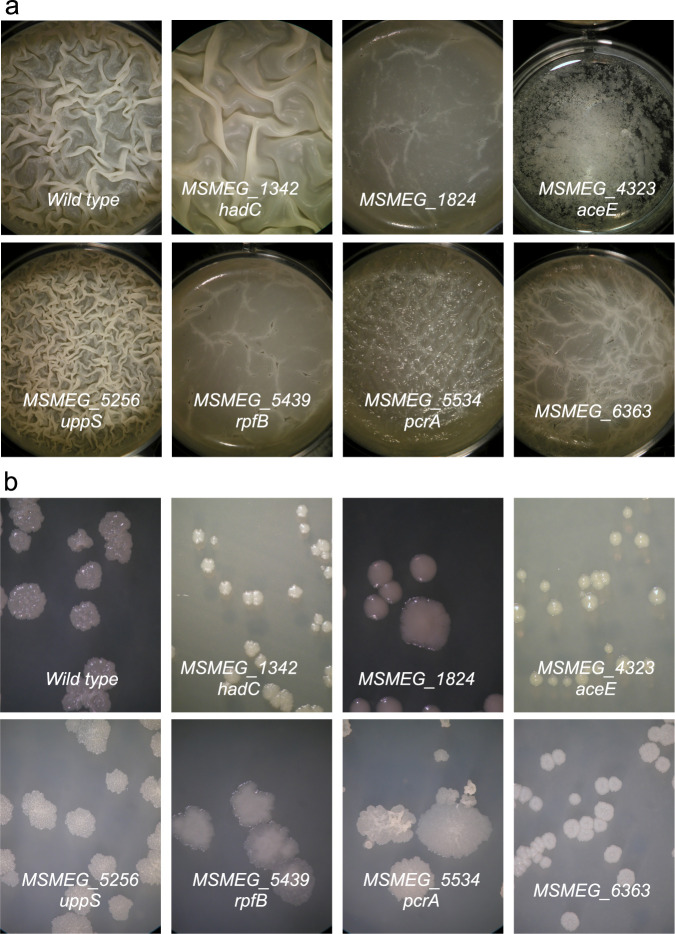
Biofilm (a) and colony morphotypes (b) of the indicated deletion mutants after 7 days of biofilm formation or 4 days of colony formation.

As an example of how this knockout collection can be used, we screened for mutants that are defective in biofilm formation. Strains were inoculated into liquid biofilm medium, and biofilm formation was assessed visually for up to 10 days of growth. Seventeen candidates were identified from an initial library of 468 deletion mutants. Of these, 9 had a reproducible, altered biofilm phenotype on subsequent screens (Table 1). The mutant phenotypes had one of three morphotypes (Fig. 2a): (i) hyper-pellicle formation (e.g., Δ*MSMEG*_5256), (ii) smooth pellicle with no or little architecture (Δ*MSMEG_1824*), or (iii) no biofilm (Δ*MSMEG_4323*). In addition to the biofilm morphotype, the colony morphology of each of the mutants was determined (Fig. 2b). Again, distinct and reproducible changes in colony morphology were observed, although two strains (Δ*MSMEG_2760* and Δ*MSMEG_5487*) retained the wild-type colony morphotype (Table 1). Thus, through this simple screen we identified 9 genes that alter biofilm/colony morphology by either directly or indirectly altering the cell wall composition. As an independent validation of this screen, mutants of three of the genes had been previously associated with an altered biofilm. A transposon insertion in *MSMEG_4323* was identified because it caused an unusual colony morphology; this mutant was subsequently shown to form defective biofilms (38). *MSMEG_5439* encodes resuscitation promoting factor B (*rpfB*); when *rpfB* and *rpfA* (a paralog) mutants are combined, the resulting strain has altered colony and biofilm morphologies (39). Deletion of the M. tuberculosis *hadC* gene (Rv0637; *MSMEG_1342* homolog shares 68% amino acid identity) reduced biofilm thickness and resulted in the loss of extra-long mycolic acids from cell wall fractions (40). Thus, the phenotype of the orthologous M. smegmatis *hadC* knockout (Δ*MSMEG_1342*) reproduces that described in M. tuberculosis, further demonstrating the potential of this type of screen to provide insight into other mycobacterial species.

**TABLE 1:** Summary of biofilm and colony phenotypes of knockout mutants screened for altered biofilm formation^*a*^

MSMEG_gene	Gene name	Putative function	Colony morphology	Biofilm morphology compared with wild type
1342	*hadC*	ACP dehydratase	Flatter smoother	Delayed formation, less developed architecture
1824	*-*	LytR family transcriptional regulator	Flat, smooth, round shape	Smooth, no architecture
2760	*-*	Polyphosphate glucokinase	Similar to wild-type	No biofilm
4323	*aceE*	Pyruvate dehydrogenase	Cream colored, smooth surface, round shape	No biofilm
5256	*uppS*	UDP diphosphate synthase	Flat with pebbled surface	Hyper-pellicle, extensive architecture
5439	*rpfB*	Resuscitation-promoting factor	Smooth, flat, irregular shape	Smooth, no architecture
5487	*-*	Sensor histidine kinase	Similar to wild-type	Smooth little architecture
5534	*pcrA*	ATP-dependent DNA helicase PcrA	Flat, bumpy surface, irregular shape	Smooth little architecture
6363	*-*	Cysteine desulfurase	Whiter, smooth, flat doughnut surface	Smooth, no architecture

a See Fig. 2a and b for representative examples of different morphotypes. The symbol “-” indicates no gene name has been assigned to date.

### Generation of a CRISPRi library of essential genes.

Defining functions for essential genes requires the ability to either conditionally repress or express the target gene. Here, we took advantage of the CRISPRi system optimized for mycobacteria, which allows gene-specific transcriptional repression (41). A total of 843 genes were targeted for transcriptional repression by CRISPRi. These included highly conserved essential genes from the MSR list, highly conserved orthologs that are essential in M. smegmatis or M. tuberculosis (28) and are not in the MSR gene list because they are not sufficiently conserved in all five mycobacterial species, and all genes for which a knockout clone was not obtained (Tables S1 and S3). We note that during the course of this study, a similar arrayed CRISPRi library targeting 272 essential M. smegmatis genes with direct M. tuberculosis orthologs was also generated (42). The MSR collection includes plasmids for all but 14 of the 272 essential genes targeted and, thus, this collection extends that library for CRISPRi applications in mycobacteria. Each gene sequence was screened to identify the optimal protospacer adjacent motif (PAM) guide sequences (5′-AGAAW-3′). Small guide RNAs (sgRNAs) were then designed 5′ to the identified PAM.

10.1128/mBio.02401-20.7 TABLE S3 List of CRISPRi clones with sgRNA targets. Download Table S3, XLSX file, 0.05 MB. Copyright © 2021 Judd et al. 2021 Judd et al. https://creativecommons.org/licenses/by/4.0/ This content is distributed under the terms of the Creative Commons Attribution 4.0 International license.

### Testing CRISPRi efficacy of essential genes by viability assessment.

**FIG 3:**
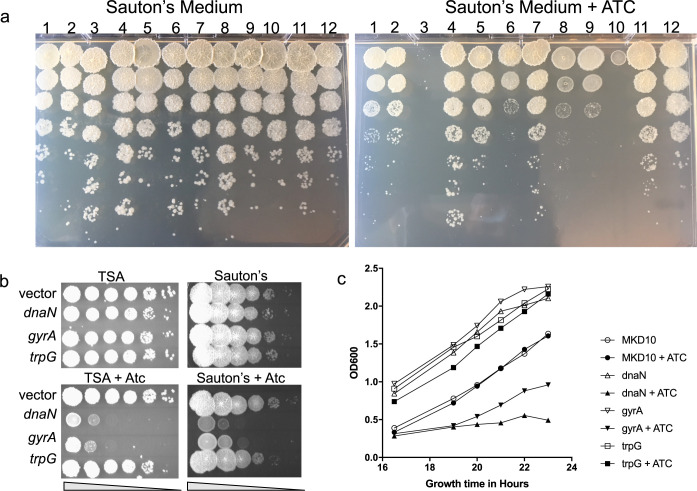
Growth defects caused by expression of CRISPRi sgRNAs. (a) M. smegmatis containing pJR962 derivatives expressing sgRNAs targeting individual genes were grown to stationary phase in TSB in the absence of Atc. The cells were serially diluted 10-fold and 5 μl of each dilution was spotted onto Sauton’s medium without (left) or with (right) Atc, which induces expression of the CRISPRi system. The strain in column 5 is the control, wild-type mc^2^155, not containing a pJR962 clone. Genes targeted in each column are from left to right: 1*-MSMEG_0029*, 2-*0244*, 3-*0317*, 4-*0482*, 6-*0709*, 7-*0789*, 8-*0832*, 9-*0956*, 10-*1019*, 11-*1066* and 12-*1214.* (b) Three clones (*dnaN*, *gyrA*, and *trpG*) were selected for further analysis. Cultures were diluted and spotted onto TSA or Sauton’s medium with or without Atc and growth was compared. The effect of repressing *trpG* can be seen in the more minimal Sauton’s medium. (c) Growth curves of cultures grown in TSB with or without Atc provide a more quantitative assessment on overall growth. MKD10 is the mc^2^155 parental control containing the empty vector pJR962. All cultures were diluted 100-fold before addition (or not) of Atc; the first readable data point was following overnight growth at 16.5 h.

To test the effect of CRISPRi on cell growth, 82 plasmids from a single 96-well plate were introduced into mc^2^155 by electroporation and individually tested by spotting cells on rich medium (TSA) with and without anhydrotetracycline (± Atc). Using a simple endpoint assay, 25/82 of the clones were completely inhibited for growth, while another 19 exhibited reduced colony size, consistent with repression of a gene that is either essential or is required for optimal growth under that condition (data not shown). The sensitivity of the spotting assay was further increased by spotting dilutions of cells onto defined Sauton’s medium (± Atc). Serial dilutions allowed for better side by side comparisons, including the effect of gene repression on colony size and nutritional requirements (Fig. 3a). Inhibition of two genes in the panel, *MSMEG_0317* and *MSMEG_1019*, abolished growth (columns 3 and 10), while inhibition of *MSMEG_0709* (*dnaK*), *MSMEG_0832* (*def*), and *MSMEG_0956* (*hemB*) severely reduced viability (columns 6, 8, and 9). This is in agreement with Tn-seq predicted growth-defect/essentiality calls (28). In these preliminary screens, ∼60% of transformants exhibited reduced growth on Sauton’s medium, further underscoring the efficacy of the CRISPRi system. One notable benefit of CRISPRi repression is that it can target multiple gene copies. *MSMEG_1019* (Fig. 3a, column 10) is in a duplicated region of M. smegmatis (*MSMEG_0991-1044* and *MSMEG_2285-2337*) and, thus, its function cannot be determined by Tn-seq or single-gene knockout. However, the CRISPRi sgRNA also represses the second gene copy, *MSMEG_2299*, and indicates they encode an essential gene product. Their ortholog in M. tuberculosis, *Rv3053* (*nrdE*), is essential (43). Indeed, we have observed similar inhibition with CRISPRi clones targeting *MSMEG_1017* (*nrdH*) and *MSMEG_1033* (*nrdG*) in this same region, further underscoring the potential of CRISPRi inhibition (data not shown).

Three gene targets were selected for further analysis: *dnaN*, *gyrA*, and *trpG* (*MSMEG_0001*, *MSMEG_0006*, and *MSMEG_0029*, respectively). A dilution series on solid rich medium indicated that *dnaN* and *gyrA* were essential for growth, while *trpG* was not essential in rich medium (± Atc, Fig. 3b). The *trpG*-targeted strain was shown to be slightly growth-inhibited on Sauton’s medium with Atc, indicating the condition-specific function of this gene (Fig. 3b). Growth curves of strains containing these three clones in liquid medium compared to wild-type mc^2^155 also showed that the expression of the CRISPRi clone targeted to *dnaN* and *gyrA* suppressed growth (Fig. 3c). The liquid growth assay provides a more quantitative view of suppression and identifies the window in which the repressed gene has the largest impact on relative growth rates (±Atc). We expanded the growth assays to a 96-well plate format and found that the relative growth rates (± Atc) were largely consistent with the spot endpoint assay (Fig. S3). However, observed growth inhibition differences dependent on the time of incubation with Atc suggest that the effects of CRISPRi-mediated suppression of some genes will be growth-rate dependent, presumably reflecting protein turnover and dilution following cell division. We envisage the 96-well assay providing a high-throughput format that can use different media and different cell stresses to reveal conditional sensitivities caused by induced repression of the target gene. Moreover, the plasmid resource can be introduced into other M. smegmatis derivatives, such as a specific gene knockout strain, to identify synthetic genetic connections obscured by redundant or compensatory pathways in a wild-type environment.

10.1128/mBio.02401-20.3 FIG S3 Relative growth of M. smegmatis containing different CRISPRi clones grown in 96-well plates. Cells were inoculated into TSB (± Atc at 50 ng/ml) in 96-well plates and shaken at 37°C, with intermittent OD_600_ measurements in a microtiter plate reader. The data are plotted as the growth differential (OD_600_ with Atc − OD_600_ without Atc) over time (hours). Three representative sets of 11 clones are shown for simplicity. The names of the genes targeted for suppression are shown to the right of each graph. Most CRISPRi clones caused a reduction in relative growth, although some failed to effect growth in these rich-media conditions. We note that in this particular set of experiments, the CRISPRi plasmid targeting *MSMEG_0001*(*dnaA*) efficiently suppressed growth until the 62 h time point, which was followed by a steady increase in OD_600_ (middle panel). We believe this is growth of a contaminant, as this is an essential gene and we see no growth in other experiments using this CRISPRi plasmid. Download FIG S3, TIF file, 0.8 MB. Copyright © 2021 Judd et al. 2021 Judd et al. https://creativecommons.org/licenses/by/4.0/ This content is distributed under the terms of the Creative Commons Attribution 4.0 International license.

While we have not independently validated all 843 CRISPRi clones for their ability to silence genes, our preliminary data indicate that ∼60% will prevent or retard growth in standard culturing conditions. The lack of growth inhibition with some of the clones could be due to multiple reasons, including that knockdowns reduce target protein production as opposed to elimination by gene knockouts, that the gene targeted is essential only under specific conditions not tested here, that the gene was originally misassigned as essential, or that the gene was targeted by CRISPRi because it was refractory to conventional knockout. We used quantitative reverse transcriptase PCR (qRT-PCR) to verify CRISPRi inhibition in a subset of targeted genes (Fig. S4). We consistently saw a reduction of steady-state levels of mRNAs for the targeted genes upon CRISPRi induction, showing the CRISPRi system was functioning as intended, even in strains that were not growth inhibited. The qRT-PCR assay may be useful for individuals to assess CRISPRi efficacy provided that their gene of interest is reasonably well transcribed and does not have overlapping antisense transcripts, which the primers will also amplify. Culture conditions, including medium composition, nutrient restriction, antibiotic exposure, temperature, or many other extrinsic parameters may reveal genetic dependencies not observed in our cursory test.

10.1128/mBio.02401-20.4 FIG S4 Quantitative reverse transcriptase PCR (qRT-PCR) confirmation of transcriptional repression by CRISPRi. Four derivatives of mc^2^155 were selected, which encoded CRISPRi-sgRNA targeting four genes: *MSMEG_0005, 0952, 0974*, and *0457* (encoding gyrB, hemA, ccsB, and TopoIVB, respectively). Cultures were grown to an OD_600_ of 0.5 before addition of Atc to 500 ng/ml. Following 3 h of treatment, cells were harvested and RNA purified using Trizol and bead beating according to the manufacturer’s instructions (Ambion-Life Technologies). The presence of contaminating DNA was eliminated by DNaseI digestion and verified by negative-end-point PCR with appropriate controls prior to reverse transcription. qRT-PCR was performed using gene-specific primers according to the manufacturer’s instructions (Applied Biosystems). Amplification was quantified using SYBR Green in an Applied Biosystems 7500 RT PCR machine. The fold change in mRNA was normalized with respect to *rpoB* mRNA and is shown in the graph relative to the unrepressed gene under each condition (± Atc). All four genes exhibited similar levels of transcriptional repression. In plating assays, strains targeting the three essential genes (*gyrB, hemA,* and *ccsB*) exhibited no growth or reduced viability (data not shown), consistent with CRISPRi repression. Msmeg_0457 is nonessential under lab conditions and, as expected, when targeted by CRISPRi cells grew similarly to the wild-type control (data not shown). Download FIG S4, TIF file, 0.8 MB. Copyright © 2021 Judd et al. 2021 Judd et al. https://creativecommons.org/licenses/by/4.0/ This content is distributed under the terms of the Creative Commons Attribution 4.0 International license.

### Generation of a plasmid library of gene fusions for protein localization studies.

**FIG 4:**
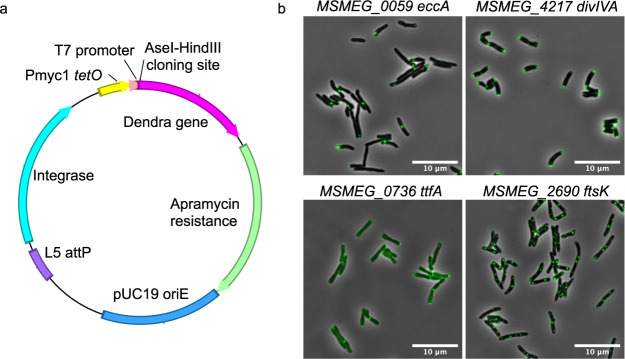
(a) Schematic representation of the vector used for expression of Dendra gene fusions. Digestion with AseI and HindIII facilitates InFusion cloning of open reading frame amplicons, disrupting the ATTAAT AseI site to ATTAtg to form an ATG initiation codon for ORF expression. The AAGCTT HindIII site is regenerated and replaces the native ORF stop codon to allow continued translation into the alanine/glycine linker and Dendra. (b) Examples of protein localization to poles or septa with different Dendra fusion proteins expressed in M. smegmatis. Gene numbers and name are indicated.

Determining the localization of a protein within a cell can provide important insights into its function, especially if colocalization occurs with proteins of known function. We therefore created a library of fusions to the fluorescent protein Dendra (a monomeric, green-to-red photoactivatable monomeric fluorescent protein [44]), in order to both image the location of individual proteins and facilitate analysis of their colocalization with other core proteins. Individual core genes were PCR amplified with a high-fidelity DNA polymerase such that the amplicon ends were compatible with ligation-independent cloning. A plasmid was optimized for high-throughput gene cloning and controlled gene expression in mycobacteria (Fig. 4a). Briefly, the plasmid has a pUC origin of replication to allow growth in E. coli, and it encodes resistance to apramycin (Apr^r^). It integrates site-specifically into the M. smegmatis chromosome at the L5 phage *attB* site, which ensures stable maintenance and a single-gene copy (45). The cloned gene is transcribed from the characterized, constitutive mycobacterial promoter P_smyc_, which includes TetR operator sites to allow the option of precise control of gene expression via the Tet repressor protein (46). In addition, the plasmid provides T7 promoter and terminator sequences that allow overexpression in E. coli or M. smegmatis strains expressing T7 RNA polymerase. Unique restriction sites allow cloning in-frame with the *dendra* gene, connected via a flexible poly-alanine-glycine linker. The *dendra* gene was codon optimized for expression in mycobacteria and includes a C-terminal FLAG tag to also allow affinity purification or coimmunoprecipitation (co-IP) to screen for protein interactions (Fig. 4A). Restriction sites flanking the *dendra* gene allow customization of alternative C-terminal fusion tags of an MSR plasmid construct.

A total of 1,116 genes were successfully cloned as a gene fusion with *dendra* (Table S1). Each cloned gene was sequence-verified using plasmid-based sequencing primers flanking the cloning site to read in from the 5′ and 3′ ends of each inserted MSR gene. Sanger sequence reads average 700 nt; thus, genes greater than ∼1.4 kb in length were not completely sequenced. Only two PCR-based mutations were detected in all sequence reads, consistent with the high fidelity of the Q5 DNA polymerase (New England BioLabs [NEB]). Thus, this low mutation rate indicates that undetected mutations are extremely unlikely in the longer genes.

### Visualizing Dendra fusion protein localization.

While the cloned library of genes has multiple uses, we chose to use it to determine protein localization by fluorescence microscopy. We examined the protein localization of 1,043 gene fusions in M. smegmatis and acquired over 7,000 high-quality fluorescent images. Of these, 761 strains yielded over 100 high-quality segmented single cells using a customized image analysis pipeline. The images indicated that many of the proteins have reproducible, characteristic positions in the mycobacterial cell, including polar, peripolar, envelope, mid-cell, septal, and as discrete cellular foci (Fig. 4b). Each of these locations suggests functional roles consistent with subcellular structures or regional multiprotein factories. Representative images for all gene fusions are available for viewing on our bioinformatics resource web site, along with summary plots of protein localization, heat maps, and the impact of fusion expression on cell length and shape based on hundreds of images per construct (https://msrdb.org/). A more detailed description of these analyses is in preparation (Zhu, J., Wolf, I.D., Dulberger, C.L., Rubin, E.J., and Fortune, S.M.).

As expected, some patterns of protein localization were consistent with those previously described in both M. smegmatis and M. tuberculosis. For example, proteins of the ESX-1 secretion system are known to be polar localized (47, 48). A Dendra fusion of the Esx1 protein EccA was polar localized (Fig. 4b). DivIVA is an essential protein in mycobacteria, as it plays a fundamental role in recruiting enyzmes required for cell wall synthesis at the cell pole (49). Our fluorescent images confirmed the extreme polar localization of DivIVA. Cells expressing DivIVA-Dendra were also misshapen and short, consistent with a dominant negative effect of expressing a properly folded fusion protein interfering with normal cell wall synthesis (Fig. 4b). In agreement with previous studies, we identified MSMEG_4287, MSMEG_0736 (TtfA), MSMEG_2690 (FtsK), and MSMEG_0035 (FhaA) as localized to the poles and septum (50–52) (Fig. 4b, and web site).

**FIG 5:**
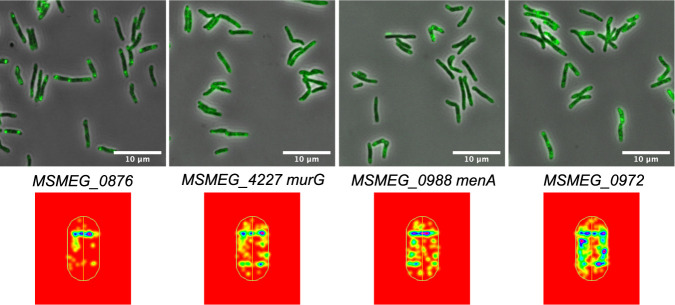
A unique class of proteins is localized to the intracellular membrane domain (IMD) (53). Each Dendra fusion protein is shown expressed in M. smegmatis and characteristically features two prominent patches at the cell pole (top panel). The heat map below visually reinforces the similarity of protein localization for each fusion protein. The heat map is a representative cell with consolidated mapping data for 50 to 100 cells. Red indicates no fluorescence, while increasing intensity and protein localization is reflected by changes from yellow to green, blue, and purple. There were ∼30 protein fusions that had similar localization patterns. Gene numbers and name (if known) are given.

Colocalization of proteins in a cell often indicates proteins are interacting as a part of the cellular machinery, either directly or indirectly. The availability of a library of fluorescent images offers the ability to screen for similar patterns, which would be indicative of proteins colocalizing to the same site(s) in the cell. In our initial analysis, we identified a class of ∼30 proteins that had very similar patterns, in which two patches of fluorescent protein are located close to a pole (Fig. 5, e.g., MSMEG_0876). This was especially apparent using the heat maps generated from consolidated mapping data for 50 to 100 cells. Remarkably, many of these proteins had been previously identified by mass spectrometry as components of the intracellular membrane domain (IMD) (53). The IMD is concentrated in the polar region of growing cells and includes many proteins required for synthesis of cell envelope components (e.g., PimB’, MSMEG_4253, and MurG, MSMEG_4227) (Fig. 5). Subsequent analyses have confirmed that at least three of the colocalizing proteins are associated with the IMD, while the remaining seven are still under investigation (MSMEG_0988 [MenA] and MSMEG_0972) (Fig. 5) (54 and Y. Morita personal communication.). Thus, identification of similar protein localization patterns has independently validated the association of these proteins within the IMD and has identified additional putative IMD proteins that escaped detection by mass spectrometry. Many of these proteins are essential in both M. smegmatis and M. tuberculosis; hence, they likely play similar fundamental roles in cell wall precursor synthesis in all mycobacteria.

## DISCUSSION

We have created a resource for the mycobacterial community by focusing on a set of 1,153 proteins that are highly conserved across all mycobacterial species (Fig. 1b). This level of conservation ensures that these proteins mediate fundamental mycobacterial processes, and that any genetic, phenotypic, and bioinformatic data generated from their analysis will be applicable to other species of mycobacteria. These insights will open new avenues of research into the less experimentally tractable pathogens. The current main mycobacterial genetic resource, BEI Resources, contains no mutants or cloned genes of any mycobacterium other than M. tuberculosis (M. Hazbón, personal communication). By selecting the most conserved genes, we have streamlined more comprehensive genome-wide approaches to focus on core gene functions applicable to all species and, thus, enhanced our ability to characterize other important nontuberculosis mycobacteria (including M. abscessus, M. avium, M. kansasii, M. leprae, M. marinum, and M. ulcerans). We believe this collection will provide an experimental foundation to verify existing putative functional annotations or initially assign function to genes that currently lack direct empirical support. Importantly, 60% (696) of the genes have no assigned locus name, underscoring the gap in our knowledge of mycobacterial biological processes and the potential of this resource.

A dedicated website has been created (https://msrdb.org/) that lists all genes and vectors in the MSR collection, along with downloadable files describing the different clones available. There are representative images of cells expressing the Dendra fusions for each gene, including a preliminary assignment of fusion protein localization, heat maps, and observed cell growth effects resulting from ectopic expression. The searchable data allows subsets of genes to be compared, as well as individual gene pages. The individual plasmids and strains will be available to all researchers through Addgene.

The collection was produced with measures to minimize secondary mutations and other effects, such as gene polarity or poor expression. While we cannot rule out extragenic suppressor mutations, or downstream polar effects of gene knockouts, for all clones, we believe that the combined application of different components of the collection will allow researchers to focus on defining gene function. For example, a phenotype associated with a gene in a large operon could be dissected further using additional CRISPRi or gene knockouts already available in the MSR, while Dendra fusions of the different operonic genes could be used for complementation studies and to determine if the proteins colocalize, consistent with a common function and phenotype. Independent mutations would need to be made to rigorously confirm any novel phenotype, but the combined components of the MSR represent a first step toward establishing a mycobacterial “ecocyc” resource.

The arrayed knockout and CRISPRi libraries can be applied to high-throughput, phenotypic assays (e.g., growth rates, drug susceptibility, or morphotypes) to provide insights into gene function, as we described in a screen for biofilm mutants. As an example of the power of these broader unbiased approaches, a recent and elegant study combined CRISPRi arrays to suppress transcription of essential M. smegmatis genes, and live imaging to identify distinct cell morphotypes associated with essential gene suppression that could be correlated with known gene functions (42). We envision that the MSR CRISPRi collection will add to these and similar studies. The collection of gene clones in the *dendra* expression vector has multiple purposes beyond mapping protein localization. A gene fusion can be transformed into a knockout strain to assess complementation of a phenotype. The L5 *attB* integration site is widely conserved in mycobacteria, which could facilitate the use of individual MSR Dendra plasmids, or the entire arrayed library, into other mycobacterial species. The gene fusion can be overexpressed by T7 RNA polymerase in E. coli, and the protein can be affinity purified using the FLAG-tag epitope. The epitope can also be used for coimmunoprecipitation (co-IP) experiments in mycobacteria to screen for protein-protein interactions. Expression in mycobacteria can be controlled by introducing TetR coexpression, with regulation by anhydrotetracycline (Atc). Lastly, we expect further analysis of the fluorescence microscopy data will allow us to assign functions to uncharacterized proteins. We believe that further analyses of these images in combination with the knockout and CRISPRi libraries will dramatically enhance mycobacterial research, especially in assigning functions to the many hypothetical open reading frames found in mycobacterial genomes.

## MATERIALS AND METHODS

### Bacterial strains and cultures.

M. smegmatis wild-type strain mc^2^155 and its derivatives were grown in tryptic soy broth + 0.05% Tween 80 (TSBT) or on TSA plates, and cultured at 37°C. Antibiotic selection for reporter maintenance or mutation selection strategies included apramycin (12.5 μg/ml on agar, 10 μg/ml in broth), hygromycin (100 μg/ml and 25 μg/ml, respectively), kanamycin (50 μg/ml and 10 μg/ml, respectively), and zeocin (50 μg/ml and 25 μg/ml, respectively). Escherichia coli Stellar (Clontech) and NEB5-alpha (New England BioLabs) cells were used for transformation for all plasmid constructions using the Inoue method (55).

### Primer design.

All sequence-based decisions used the reference M. smegmatis genome (CP000480.1). For each gene knockout, seven oligonucleotide primers were used that enabled arm amplification, sewing, and confirmation of the knockout mutation (Fig. S2 in the supplemental material). Primers were designed to avoid primer-dimer formation, and with optimal annealing temperatures of ∼65°C. Deletions were created inside the target gene, where the deletion extended from the second codon to 10 amino acids before the C terminus and stop codon. For expression plasmids, primer sequences for cloning individual ORFs into pMSR3 were designed to amplify each gene from the second to the penultimate codon; this ensured in-frame cloning with the vector-based features (e.g., ribosome-binding site, poly-glycine-alanine linker). All primer sequences are available upon request.

### Generating precise deletions by recombineering.

M. smegmatis cells containing pJV53 were made electrocompetent for recombineering as described (56). The recombineering substrate was made by a two-step PCR method to avoid plasmid-based, multiple cloning steps (Fig. S2). First, two homology arms (300 to 500 bp each) flanking the target gene were generated by PCR amplification, with the gene-proximal primer positioned at the deletion junction and tailed with a universal priming site in its 5′ half. These arms served as the templates for a second round of “SOEing,” i.e., overlap-extension PCR, that included a third fragment containing the gene conferring resistance to zeocin (Zeo) flanked by the universal priming sites. This second round of amplification used nested primers in the arms to drive the amplification of the intended arm-Zeo-arm recombineering substrate. The purified substrate was electroporated into recombineering-proficient cells and plated on TSA + Zeo to identify candidate mutant clones. These clones were verified by a 3-primer PCR, which contained a flanking primer capable of amplification with either a Zeo cassette-specific primer or a target gene primer. The 3-primer PCR product size allowed discrimination between the wild-type gene and its targeted replacement by Zeo. The PCR always generated a product and verified locus-specific recombination, as opposed to ectopic insertion. The Zeo-gene cassette consists of a compact constitutive promoter and the *zeo* gene, which are flanked by *loxP* sites for Cre-mediated recombination, and unique priming sites to facilitate SOE PCR. The DNA sequence and plasmid are available on request (Addgene).

### Generation of Dendra gene fusions.

An integrating plasmid, pMSR3, was created with unique restriction sites that allow cloning in-frame with the *dendra* gene via a flexible poly-alanine-glycine linker (Fig. 4A). The plasmid encodes the *aacC41* allele of a gene encoding apramycin resistance to ensure it confers resistance exclusively to apramycin (57). The *dendra* gene was codon optimized for expression in mycobacteria and includes a C-terminal FLAG tag (sequence and plasmid available on request via Addgene). The plasmid was digested with AseI and HindIII to generate appropriate overhanging ends compatible with InFusion cloning. Genes were amplified from genomic DNA using primers with 5′ ends that anneal to the cleaved ends of the vector. PCR amplification utilized a high-fidelity polymerase (NEB Q5 polymerase, New England BioLabs), with extension times adjusted for longer target genes. All steps of cloning were performed in 96-well plates. Purified PCR products were recombined with the linearized plasmid and transformed into chemically competent E. coli cells. Transformants were selected on LB agar that contained apramycin. Colonies were screened by colony PCR with flanking primers to identify clones containing inserts of the predicted size for each ORF. Plasmid DNA was then purified from positive clones and the gene sequence confirmed by Sanger sequencing using the flanking primers. In all, 1,116 genes were successfully cloned in-frame with the *dendra* gene (Table S1).

### Imaging by fluorescence microscopy.

Individual core *gene*::*dendra* fusion plasmids were electroporated into M. smegmatis mc^2^155 and transformants were selected on TSB agar that contained apramycin. Colonies were picked and cultured, arrayed, and stored in 96-well microtiter plates. As a quality control, random wells were screened by PCR from each plate to confirm each well contained a gene fusion of the correct size. Frozen stocks were grown to early stationary phase to achieve similar cell densities before cells were subcultured into replicate plates. The plates were grown with shaking at 37°C in liquid 7H9 medium consisting of Middlebrook 7H9 salts supplemented with 0.2% glycerol, 0.05% Tween 80, ADC (albumin, dextrose, and catalase), and apramycin (12.5 μg/ml). Cell cultures of optical density at 600 nm (OD_600_) of ∼1.0 to 3.0 were spotted onto 96-pedastal slides (2.5% agarose) and imaged with a Plan Apo 100× 1.45 NA objective using a Nikon Ti-E inverted, widefield microscope equipped with a Nikon Perfect Focus system with a Piezo Z drive motor, Andor Zyla sCMOS camera, and NIS Elements version 4.5. To excite the Dendra fluorophore, a Spectra X LED light source (470/24 nm) was used and paired with Sedat Quad filter sets (515/30 nm). The pedestals were maintained at 37°C using an environmental chamber, and randomized fields were acquired until approximately 100 to 300 cell images had been captured. In total, 1,116 gene fusions were screened and we captured high-quality images for 761 gene fusions, allowing quantitative determination of protein localization.

### Creating a library of CRISPRi clones targeting essential genes.

When this work was initiated, 312 genes were considered essential and 29 as domain essential (E. Rubin, unpublished data). This list was subsequently expanded to 403 genes determined essential or required for growth (28), and these were targeted for transcriptional repression by CRISPRi. In addition, genes not successfully knocked out after two rounds of recombineering were also targeted for CRISPRi-based repression. Finally, 183 highly conserved orthologs were also targeted; these are essential in M. smegmatis or M. tuberculosis (28) but are not in the MSR gene list because they are not sufficiently conserved in all five mycobacterial species (Table S1). Optimal small guide RNA sequences (sgRNA) were identified within each gene by a script that first determined optimal PAM sequences and then flanking 5′ sequences (41). Oligonucleotide templates based on these sgRNAs were PCR amplified with two flanking primers, which were designed to be compatible with InFusion cloning into BsmBI-digested pJR962 (41). All clones were verified by DNA sequence analysis. Each plasmid was arrayed in a 96-well plate and, as a further control, clones were randomly selected from individual wells and confirmed by Sanger sequencing. All primer sequences and sgRNA targets are listed (Table S3). We were unsuccessful in creating a knockout or a CRISPRi-targeting clone for four of the 1,153 core genes: *MSMEG_1672*, *1828*, *4225*, and *6327*.

### Validation of CRISPRi suppression.

One arrayed 96-well plate of CRISPRi plasmid clones was electroporated into M. smegmatis mc^2^155 and transformants selected on TSB containing kanamycin. The transformants were then seeded into a 96-well dish and grown to saturation in TSBT at 37°C. The arrayed cultures were replica pinned directly onto TSA, 7H10 medium, or Sauton’s medium with and without Atc at 50 ng/ml. Plates were incubated for 4 days at 37°C and scored. Growth curves in liquid culture (± Atc at 50 ng/ml) were performed in 96-well plates and in flasks with shaking at 37°C, with intermittent OD_600_ measurements in a microtiter plate reader or in cuvettes, respectively. Cultures were grown to an OD_600_ of ∼1.0 before diluting into fresh medium (± Atc).

### Biofilm assays.

Biofilms assays were performed in 6-well dishes using either TSB or complete biofilm medium (58). An aliquot of 10 μl of an overnight culture was used to inoculate 5 ml of medium in each well. Biofilms were then incubated for up to 10 days with minimal handling at 30°C, scored, and photographed.

### Bioinformatics.

Optimal primer pairs for amplification, cloning, and screening were generated from custom scripts written in R and these are available upon request. The website at https://msrdb.org/ was created with Python3, using Flask and SQLAlchemy. The data are stored in an underlying PostgreSQL database and the site is hosted by Webfaction.
