# Supplementary figures and images for: A Mycobacterial Systems Resource for the Research Community
Source: mBio. 2021 Mar 2;12(2):e02401-20. doi: 10.1128/mBio.02401-20 (PMC8092266; doi:10.1128/mBio.02401-20)

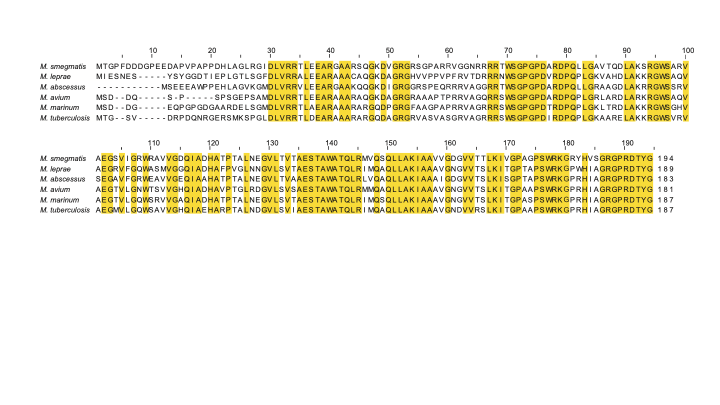

Supplement: FIG S1 [file mBio.02401-20-sf001.tif]

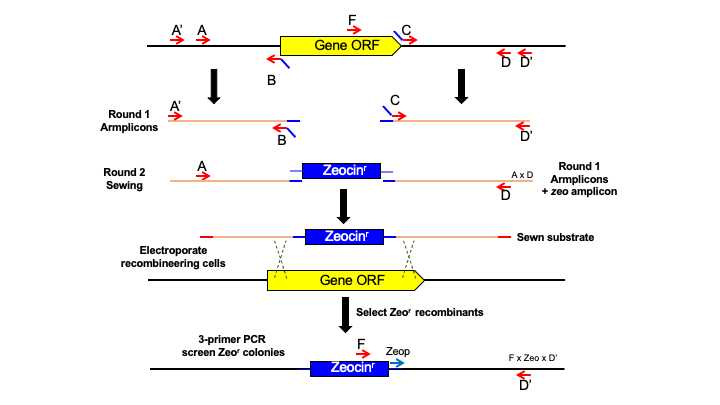

Supplement: FIG S2 [file mBio.02401-20-sf002.tif]

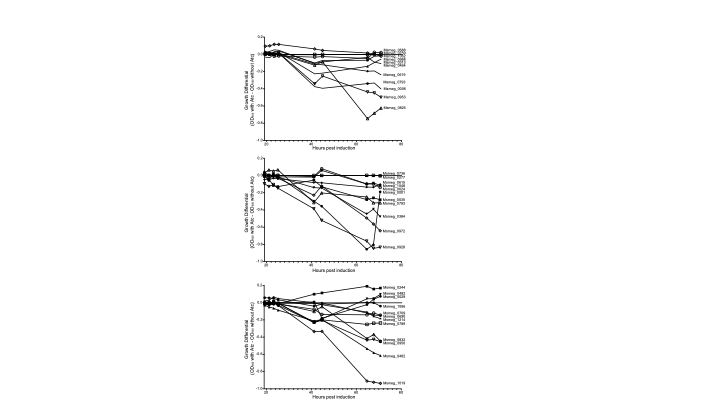

Supplement: FIG S3 [file mBio.02401-20-sf003.tif]

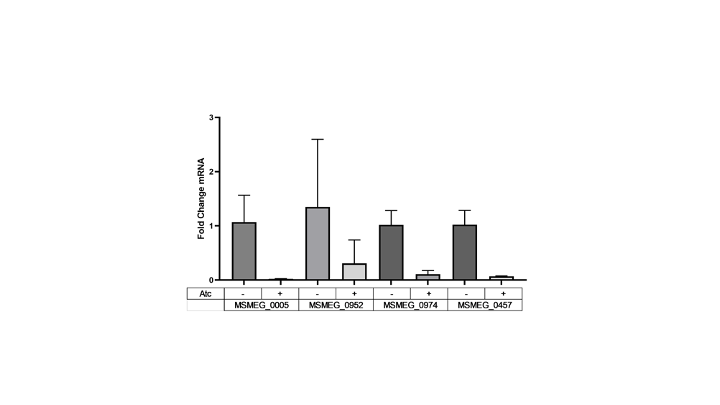

Supplement: FIG S4 [file mBio.02401-20-sf004.tif]
